# Plasticity Improvement in a Co-Rich Co_40_Fe_25_Cr_20_Ni_15_ High-Entropy Alloy via Al Alloying

**DOI:** 10.3390/ma16031149

**Published:** 2023-01-29

**Authors:** Yuxiao Li, Yu Chen, Raymond Kwesi Nutor, Nan Wang, Qingping Cao, Xiaodong Wang, Dongxian Zhang, Jian-Zhong Jiang

**Affiliations:** 1 International Center for New-Structured Materials (ICNSM), State Key Laboratory of Silicon Materials, School of Materials Science and Engineering, Zhejiang University, Hangzhou 310027, China; 2 State Key Laboratory of Modern Optical Instrumentation, Zhejiang University, Hangzhou 310027, China

**Keywords:** high-entropy alloys, deformation twinning, stacking fault energy, molecular dynamics simulations

## Abstract

The mechanical properties of high-entropy alloys (HEAs) can be regulated by altering the stacking fault energy (SFE) through compositional modulation. The Co-rich HEAs, exhibiting deformation twinning and even strain-induced martensitic transformation at room temperature, suffer from insufficient ductility at high strength. In this work, we developed Co-rich (Co_40_Fe_25_Cr_20_Ni_15_)_100−x_Al_x_ (x = 0 and 5 at.%) HEAs and investigated their tensile behaviors at room temperature. The addition of Al resulted in a massive improvement in the strength-ductility product, even at similar grain sizes, and also altered the fracture mode from quasi-cleavage to ductile dimple fracture. Interestingly, both alloys were deformed by mechanical twinning, which was also verified by molecular dynamics (MD) simulations. The MD simulations revealed the SFE increased upon Al addition; however, the slip energy barrier was reduced, which favored the mobility of dislocations and twinning propensity to prolong strain hardening. The present findings provide further insights into the regulation of mechanical properties of HEAs by Al-alloying.

## 1. Introduction

High-entropy alloys (HEAs), known as multiprincipal element alloys with individual atomic concentrations of at least 5 at.%, have gained tremendous research interest since their inception in 2004 [1,2]. Amongst them, the face-centered cubic (fcc) class of these HEAs has been extensively studied due to their impressive strength-ductility combinations, especially in cryogenic conditions [3,4]. Most notable is the equiatomic CrMnFeCoNi (so-called Cantor) alloy, which displays strength-ductility enhancement as temperature decreases as a result of the transition in deformation behavior from dislocation slip at room temperature to mechanical twinning at cryogenic temperatures [5]. This is because the stacking fault energy (SFE) is a major factor that determines the prevalent deformation mechanism at a specific temperature [6]. Apart from the temperature dependence of the SFE, it is also compositionally dependent [7]. Elements such as Co and Cr decrease the SFE while Fe and Ni increase the SFE [6,7,8]. Hence, this is the inspiration behind the development of Fe-rich [9,10] and Co-rich [11,12] HEAs, which can access twinning-induced plasticity (TWIP) and transformation-induced plasticity (TRIP) deformation mechanisms.

Indeed, these Co-rich HEAs have been primarily modeled after the Cantor alloy and its lower entropy derivates, targeting switching between the TWIP and TRIP effects [11,12]. However, the Co-rich HEAs with early access to the fcc → hexagonal close-packed (hcp) martensitic transformation suffer from insufficient ductility at high strength because of the lack of operative slip systems of the hcp phase [12]. Thus, the TWIP Co-rich HEAs with excellent strength–ductility combinations can be considered the rather attractive option, which is the aim of this work.

In several fcc-structured HEAs, Al alloying has been a commonly exploited strategy to improve the room temperature yield strength and hardness [13,14,15,16,17,18]. Without exceeding the solid-solution limit of the fcc phase, the Al addition induces a solid-solution strengthening effect due to an elastic misfit caused by an enhanced lattice distortion [17]. Meanwhile, body-centered cubic (bcc) precipitates reinforce the fcc matrix once the solute solubility is exceeded [15,19]. Furthermore, Al addition is known to modify the SFE of an alloy, which opens the avenue to accessing different deformation mechanisms [20,21]. To the best of our knowledge, the influence of Al alloying on Co-rich-type HEAs has yet to be reported. In the present work, we report the effect of Al alloying on the mechanical properties of a Co-rich Co_40_Fe_25_Cr_20_Ni_15_ HEA. The (Co_40_Fe_25_Cr_20_Ni_15_)_100−x_Al_x_ (x = 0 and 5 at.%) alloys annealed at 973–1123 K were all single fcc-structured with different grain sizes, which resulted in varying tensile responses. The deformation microstructures were then characterized by electron microscopy techniques to unravel the deformation and fracture mechanisms of the alloys.

## 2. Materials and Methods

### 2.1. Experimental

We designed and prepared two Co-rich alloys with nominal composition (Co_40_Fe_25_Cr_20_Ni_15_)_100−x_Al_x_ (x = 0 and 5 at.%) by arc-melting raw elemental pellets (Alfa Aesar > 99.8 wt.% purity) under an argon atmosphere in a water-cooled copper crucible, herein denoted as 0Al and 5Al, respectively. The ingots were cast into a 50 mm × 20 mm × 4 mm metal mold, then homogenized at 1423 K for 2 h followed by water quenching. Cold-rolling of samples was conducted with a thickness reduction of 70%, followed by recrystallization annealing at 973 K, 1023 K, and 1123 K for 2 h, and water-quenching. Hereafter, the annealed samples were denoted as 0Al-973 K, 0Al-1023 K, 0Al-1123 K, 5Al-973 K, 5Al-1023 K, and 5Al-1123 K, respectively.

X-ray diffraction (XRD) using Cu-Kα radiation was used to characterize the samples to determine the phases present. The microstructures of the samples were studied using a Zeiss Supra 55 scanning electron microscope (SEM, Zeiss, Jena, Germany) equipped with an Aztec HKL electron back-scatter diffraction (EBSD) system. The elemental distributions of the samples were also studied by SEM energy-dispersive X-ray spectroscopy (EDS). Transmission electron microscopy measurements were performed using a field-emission transmission electron microscope (FE-TEM; JEOL 2010, Tokyo, Japan) at 200 kV.

Tensile “Dog-bone” specimens with gauge dimensions of 12 × 1.18 × 2.27 mm^3^ were machined from the annealed specimens and used for the room-temperature tensile tests via a computer-controlled universal tensile testing machine (CMT5205 SANS, Shenzhen, China) at a 10^−4^ s^−1^ strain rate.

### 2.2. Computational Simulations

We performed the molecular dynamics (MD) calculations of generalized stacking fault energy (GSFE) using the large-scale atomic/molecular massively parallel simulator (LAMMPS) package [22] with an embedded-atom method (EAM) interatomic potential [23,24,25,26]. The calculation of GSFE was performed with a 5.0 × 12.3 × 8.7 nm^3^ simulation cell oriented with $\left[11\bar{2}\right]$, [111] and $\left[11\bar{0}\right]$ directions, containing 48,000 atoms. To exclude the effect of the random distribution of atoms on the GSFE, we generated 5 independent Co_40_Fe_25_Cr_20_Ni_15_ and (Co_40_Fe_25_Cr_20_Ni_15_)_95_Al_5_ models with the same composition but different atomic distributions and calculated their mean value and standard deviation. The intrinsic stacking fault was introduced by fixing the atoms in the bottom half along the [111] direction while moving the upper half of atoms along the $\left[11\bar{2}\right]$ direction. The total displacement was obtained by the Burgers vector, $B_{V}=a_{0}/\sqrt{6}$ (a_0_ is the lattice constant), of a Shockley partial dislocation. Prior to GSFE calculations, energy minimization was performed using the conjugate gradient algorithm followed by relaxation at 300 K in the isothermal–isobaric (NPT) ensemble for 80 ps. Constant temperature and zero pressure along the three directions were kept by Nose–Hoover thermostatting and barostatting [27,28]. Throughout the minimization and relaxation, periodic boundary conditions were applied in all three directions. After that, to study the effect of short-range ordering on GSFE, a 2 × 10^5^ step Monte Carlo and molecular dynamics (MC-MD) simulation was performed by randomly exchanging the atoms of different elements followed by a 1000-step MD relaxation, and the potential energy during simulations was recorded.

## 3. Results

### 3.1. Starting Annealing Microstructures

**Figure 1:**
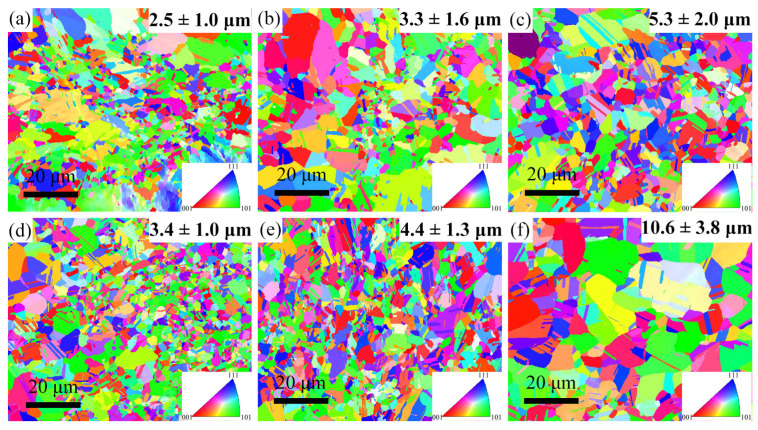
EBSD IPF maps of the (**a**) 0Al-973 K, (**b**) 0Al-1023 K, (**c**) 0Al-1123 K, (**d**) 5Al-973 K, (**e**) 5Al-1023 K, and (**f**) 5Al-1123 K specimens.

Figure 1 represents the EBSD inverse pole figure (IPF) maps of the 0Al and 5Al samples. All the samples are fcc-structured (i.e., according to the EBSD phase map, not shown here). In addition, the samples contain recrystallized grains with near-random orientations. The average grain sizes were estimated using the linear intercept method. The average grain size of the 0Al samples increased from ~2.5 µm to ~5.3 µm as the annealing temperature increased from 973 K to 1123 K. In a similar manner, the grain size increased from ~3.4 µm to 10.6 µm with annealing temperature for the 5Al sample.

**Figure 2:**
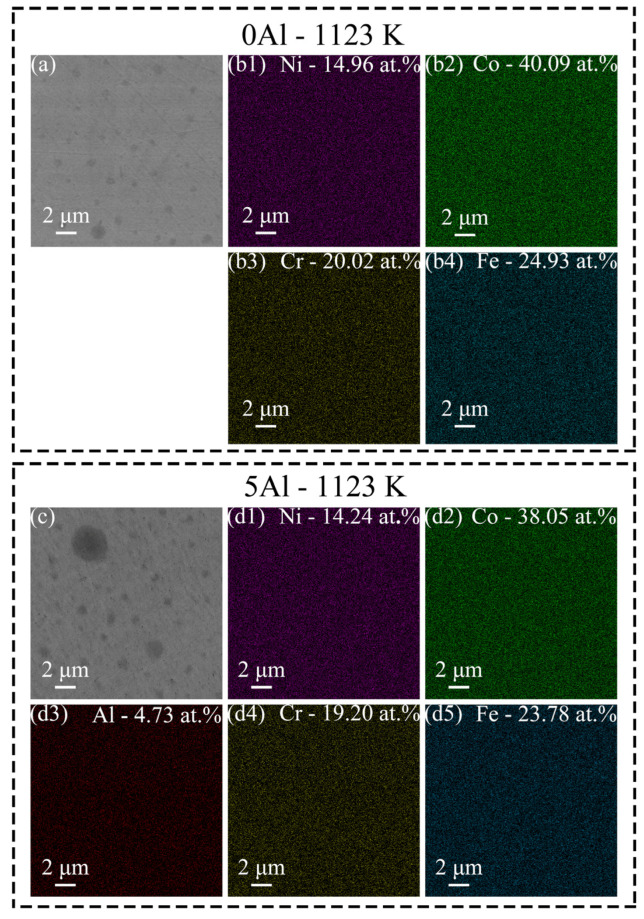
(**a**) SEM image and corresponding EDS elemental maps of (**b1**) Ni, (**b2**) Co, (**b3**) Cr, and (**b4**) Fe, respectively, in the 0Al-1123 K. (**c**) SEM image and corresponding EDS elemental maps of (**d1**) Ni, (**d2**) Co, (**d3**) Al, (**d4**) Cr, and (**d5**) Fe, respectively, in the 5Al-1123 K.

To reveal the elemental distribution within the different compositions, we performed SEM-EDS analyses on the 0Al-1123 K and 5Al-1123 K samples. Figure 2a reveals the SEM image of the region where the EDS elemental maps were taken for the 0Al-1123 K sample. It is seen that all elements are uniformly distributed without any obvious segregations (Figure 2(b1–b4)). The Co, Fe, Cr, and Ni amounts are found to be 40.09 at.%, 24.93 at.%, 20.02 at.%, and 14.96 at.%, respectively. The EDS elemental maps taken from Figure 2c show all comprising elements are homogeneously distributed in the 5Al-1123 K sample, which further confirms no segregation effect is caused by Al addition. Figure 2(d1–d5) show the elements are distributed as Co—38.05 at.%, Fe—23.78 at.%, Cr—19.20 at.%, Ni—14.24 at.%, and Al—4.73 at.%, respectively. Hence, the distribution of all elements in both samples is close to the nominal composition.

### 3.2. Tensile Behaviors

**Figure 3:**
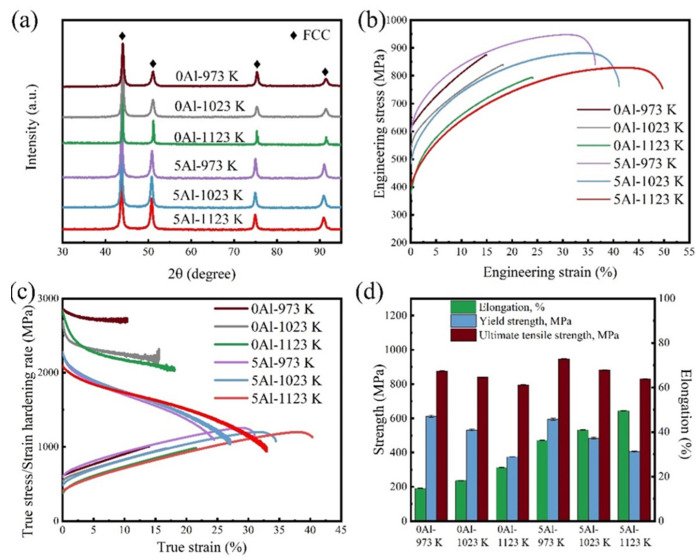
(**a**) XRD patterns; (**b**) engineering stress–engineering strain curves; (**c**) strain hardening rates and true stress–true strain curves; and (**d**) Comparison of the yield strengths, tensile strengths, and tensile elongations of the studied (Co_40_Fe_25_Cr_20_Ni_15_)_100−x_Al_x_ (x = 0 and 5 at.%) specimens.

The fcc structure of all the samples is also confirmed by the XRD patterns in Figure 3a. Thus, it is seen that no hcp phase or secondary precipitates are observed in the samples after isothermal annealing at the respective temperatures within the experimental uncertainty. Figure 3b shows the room-temperature engineering stress–engineering strain curves of the 0Al and 5Al samples. An interesting observation is the lower tensile elongations of the 0Al samples relative to the 5Al samples. The 0Al-973 K sample records the highest yield strength of 612 ± 8 MPa but the lowest fracture elongation of ~15% amongst all the samples. Meanwhile, annealing at 1123 K causes a slight improvement in fracture elongation while the yield strength is greatly reduced as seen by the yield strength and elongation of 374 ± 4 MPa and ~24%, respectively, at 795 ± 4 MPa tensile strength. The 5Al-973 K sample shows the highest ultimate tensile strength of 947 ± 2 MPa coupled with an impressive yield strength of 595 ± 7 MPa and fracture elongation of ~36%. The 5Al-1123 K shows a good combination of strength and ductility, i.e., yield strength, tensile strength, and fracture elongation of 405 ± 4 MPa, 830 ± 3 MPa, and ~50%, respectively. The yield and tensile strengths of the samples decrease while the tensile elongations increase with the annealing temperature according to the classic strength–ductility tradeoff [29]. A summary of all the mechanical properties is provided in Figure 3d. An assessment of the toughness of an alloy can be estimated as the product of the ultimate tensile strength (UTS) and fracture elongation (εf) [30]. The UTS × εf values for the 0Al-973 K, 0Al-1023 K, and 0Al-1123 K samples are 12.8 GPa·%, 15.3 GPa·%, and 19.2 GPa·%, respectively. Meanwhile, the Al-alloyed samples display massively improved strength–elongation products of 34.4 GPa·%, 36.2 GPa·%, and 41.2 GPa·%, at the respective annealing temperatures. These results demonstrate that the addition of Al positively impacts the toughness of the Co_40_Fe_25_Cr_20_Ni_15_ HEA. The strain-hardening rate (SHR) as a function of true strain for all the specimens is plotted in Figure 3c. The hardening rates of the 0Al samples are relatively higher than that of the 5Al samples. For all the samples, strain hardening is characterized by an initial rapid drop of the SHR, typical of the transition from the elastic to plastic deformation regime. Hereafter, the SHR decreases gradually with strain. The 0Al specimen failed to show any uniform elongation characteristics depicted by the absence of an intersection between the SHR and the true stress–true strain curve [31]. According to Considere’s criterion, necking is predicted to occur when the SHR < true stress for ductile materials [32]. This suggests the 0Al specimens rupture at high hardening rates without any notable necking. Meanwhile, strain hardening is prolonged in the 5Al sample, thus necking occurs at higher strains and results in better fracture elongations compared to the 0Al specimens. The significant difference in the strain hardening rates could be ascribed to different deformation mechanisms or even fracture modes in the samples [33,34].

### 3.3. Deformation Microstructures

**Figure 4:**
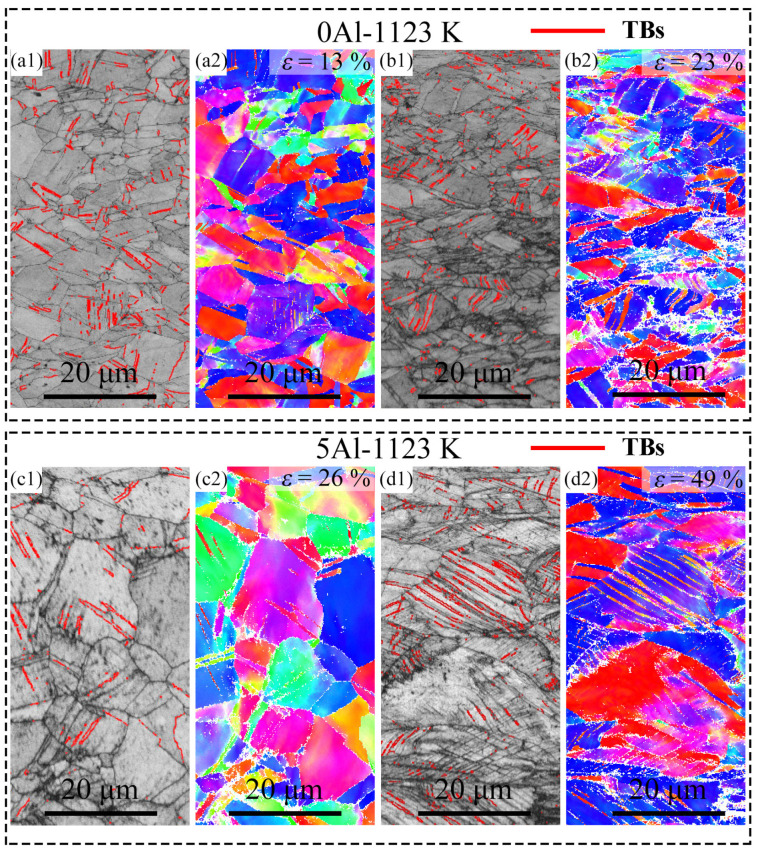
(**a1**,**b1**) EBSD IQ and (**a2**,**b2**) IPF maps of the 0Al-1123 K samples at different local strains and (**c1**,**d1**) EBSD IQ and (**c2**,**d2**) IPF maps of the 5Al-1123 K samples at different local strains.

**Figure 5:**
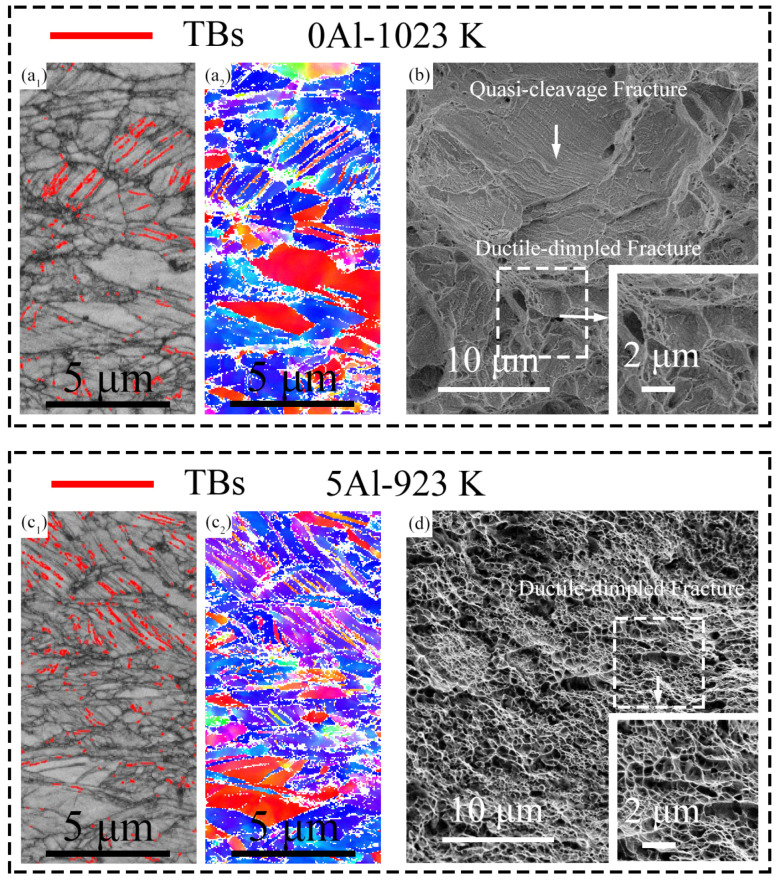
EBSD (**a1**) IQ and (**a2**) IPF maps near the fracture region of the 0Al-1023 K specimen; (**b**) SEM fracture micrograph of the tensile-fractured 0Al-1023 K specimen. EBSD (**c1**) IQ and (**c2**) IPF maps near the fracture region of the 5Al-973 K specimen; (**d**) SEM fracture micrograph of the tensile-fractured 5Al-973 K specimen.

To understand the strain hardening behavior of the samples, we observed the deformation microstructures at the different local strains in the 0Al and 5Al samples. We selected the 0Al-1123 K and 5Al-1123 K samples for observations because they displayed the optimum strength–ductility combination for their alloy classes. We note that ESBD observations of the deformed 0Al-1123 K and 5Al-1123 K samples revealed all the samples were fcc-structured and, thus, no strain-induced fcc to hcp martensitic transformation occurred. Figure 4(a1,a2) show the image quality (IQ) and IPF maps of the 0Al-1123 K samples at 13% local strain. It is seen that deformation twins (i.e., parallel lines with 60° misorientations) are formed inside the grains at this strain. However, the amount here is relatively low, such that they are sparsely seen on the IPF map. At 23% local strain (Figure 4(b1,b2)), the twin boundaries are significantly higher, hence they are readily seen in the IPF map. Meanwhile, the 5Al-1123 K sample (Figure 4c,d) also shows similar deformation characteristics. The twin boundaries at 26% local strain are barely visible in the IPF map (Figure 4(c2)) due to the limited amount but become more visible in the IPF map (Figure 4(d2)) at 49% local strain in the IPF map when they increase in amount. The observation of deformation twins at low and high strains in both samples suggests deformation twinning contributes to strain hardening in both alloys. Deformation twinning is known to be affected by the grain size of metals [35]. The critical twinning stress is higher at smaller grain sizes, which makes twinning relatively difficult in refined microstructures than in coarse grains [36]. We thus compared the number fraction of the detected twin boundaries of the IPF maps at 23% and 49% local strains of the 0Al-1123 K and 5Al-1123 K samples, respectively. The twin fractions for the 0Al-1123 K and 5Al-1123 K samples are found to be ~9.2% and ~15.3%, respectively. This indicates that mechanical twinning is more profuse in the coarse-grained 5Al-1123 K sample compared to the finer 0Al-1123 K sample. Of course, such an assessment is tricky because the dissimilar grain sizes are from different alloy compositions. To establish a better understanding, it is preferable to compare the mechanical twinning behavior of different alloy systems with similar grain sizes. From the EBSD-IPF maps in Figure 1, the average grain sizes of the 0Al-1023 K and 5Al-973 K are similar. The EBSD IQ and IPF maps at the near-fracture area of the 0Al-1023 K and 5Al-973 K samples are shown in Figure 5a,c, respectively. Again, many parallel lines are clearly visible in the grains of both samples, indicating deformation twinning is an active deformation mechanism. The number of twins is ~5.3% in the 0Al-1023 K sample and ~7.1% in the 5Al-973 K sample. This discrepancy suggests that twinning is likely prolonged in the 5Al samples, resulting in improved ductility.

Figure 5b shows the SEM fractography of the 0Al-1023 K specimen. The specimen shows a mixture of quasi-cleavage and ductile-dimple fractures. However, the quasi-cleavage mode occupies a larger area and consists of many shallow river-like lines. Such a quasi-cleavage fracture mode typically reduces the fracture resistance of alloys [37], hence the lower fracture elongations of the 0Al specimens. The 5Al-973 K specimen (Figure 5d) consists of only ductile dimples. The dimples occupy the full area of the fracture surface and are approximately several hundred nanometers in size. This ultrafine dimple size indicates that the coalescence of the micro-voids during the nucleation and propagation of a crack in the sample is significantly slow to delay fracture [34]. The appearance of the ductile-dimple fracture mode without any quasi-cleavage is responsible for the improved fracture elongations of the 5Al specimen.

## 4. Discussion

**Figure 6:**
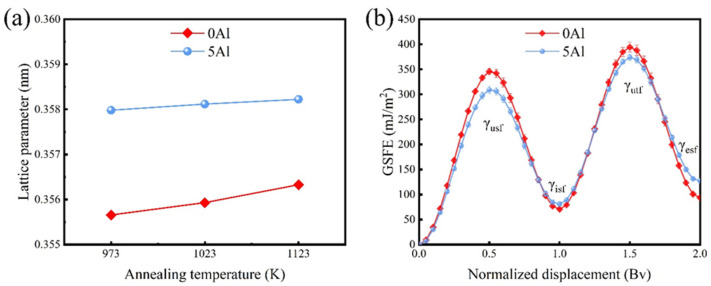
(**a**) Dependence of lattice parameters on annealing temperatures of the 0Al- and 5Al-samples; (**b**) generalized-stacking fault energy ⟨112⟩{111} slip of the 0Al-HEA and 5Al-HEA specimens at 300 K calculated by MD simulations.

Achieving an optimum strength and ductility combination is an important criterion for the design of structural materials. In the absence of forming secondary precipitates, minor alloying typically results in the improvement of the solid-solution strength (σ_s_) due to the large strain mismatch caused by the introduction of larger- or smaller-sized atoms (i.e., lattice distortion effect) into the fcc lattice [4]. Figure 6a shows the calculated lattice parameters from the XRD patterns (Figure 3a) for the 0Al and 5Al samples. It is seen that the addition of Al to the model Co_40_Fe_25_Cr_20_Ni_15_ HEA increases the lattice parameter, hence, the solid-solution strengthening by the Al is realized. According to the elasticity model for fcc HEAs, Al atoms with larger atomic sizes in the fcc lattice cause a larger lattice friction, which results in a higher strength [38].

The tensile strength and strain hardening rates are known to be highly dependent on the operative plastic deformation mechanism, which is also determined by the SFE [39]. In the currently studied alloys, we have observed deformation twinning in both the 0Al and 5Al samples, but with notably different strain hardening and fracture elongations. To understand this discrepancy, we performed MD simulations of the GSFE curves of the 0Al and 5Al samples at 300 K, as shown in Figure 6b. From the GSFE curves, the intrinsic SFE (γ_isf_), extrinsic SFE (γ_esf_), unstable SFE (γ_usf_), and unstable twinning energy SFE (γ_utf_), are calculated. Generally speaking, γ_isf_ defines the stability of a stacking fault while γ_usf_ represents the lowest energy barrier for dislocation nucleation [40]. As such, γ_isf_ is typically used to depict the SFE of an alloy system. For both alloy systems, the formation of intrinsic SFs is thermodynamically favored over extrinsic SFs since the γ_isf_ > γ_esf_. The γ_isf_ for the 0Al is found to be 70.6 mJ/m^2^ while it increases to 81.1 mJ/m^2^ for the 5Al sample. The observed significant increase in SFE upon Al alloying is expected [20,21] and is consistent with previous reports where mechanical twinning was prevalent [17,41]. It should be noted that our SFE values are relatively high compared to the proposed experimental range for mechanical twinning to occur at 15 ≤ SFE ≤ 45 mJ/m^2^ [39]. This discrepancy in experiment and theory lies in thermodynamic, magnetic, ordering, temperature, pressure, etc., factors that are difficult to replicate during theoretical calculations [42,43,44].

The question is then why do the 0Al and 5Al samples display significantly different fracture modes regardless of the mechanical twinning? To answer this, we examined the dislocation and twinning propensities of the two alloy systems. The mobility of a slip system depends on the slip energy barrier (Δγ), which is defined as the difference between γ_usf_ and γ_isf_ [45]. From Figure 6b, the Δγ_0Al_ and Δγ_5Al_ are found to be 275 mJ/m^2^ and 228 mJ/m^2^, respectively. This means that the 0Al HEA shows a higher resistance for dislocation nucleation, which is expected to affect the effective propagation of dislocations. Since the movement of dislocation is associated with plasticity, such a high-slip energy barrier indicates the mobility of dislocations in 0Al is slower, hence the lower fracture elongations of the 0Al. Furthermore, the twinning propensity in both alloys can also roughly be estimated as ${Ʌ}_{\mathrm{ratio}}=\frac{\gamma_{\mathrm{utf}}-\gamma_{\mathrm{isf}}}{\gamma_{\mathrm{usf}}-\gamma_{\mathrm{isf}}}$ [46], where a higher ${Ʌ}_{\mathrm{ratio}}$ corresponds to an improved twinning propensity. The ${Ʌ}_{\mathrm{ratio}}$ for the 0Al and 5Al samples are found to be 1.18 and 1.29, respectively. This indicates that the 5Al HEA shows a higher twinning propensity than the 0Al HEA, which is in agreement with the EBSD results (Figure 4 and Figure 5). The higher twinning propensity and lower slip energy barrier of the 5Al HEA are favorable for dislocation accumulation and mobility, which, in turn, promotes strain hardening and plasticity.

**Figure 7:**
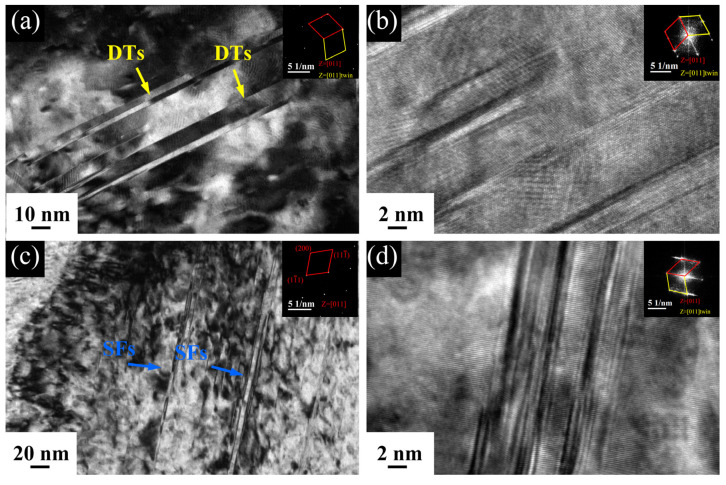
(**a**) BF-TEM micrograph showing the presence of deformation twins (inset is the corresponding SAED pattern of fcc–twin relationship); (**b**) HR-TEM image showing the fcc–twin structure; (**c**) BF-TEM micrograph of SFs (inset SAED confirms these SFs have not transformed into an hcp phase); (**d**) HRTEM of SFs in the tensile-fractured 5Al-1123 K specimen.

Considering the 5Al-1123 K sample shows the optimum mechanical properties, we performed additional TEM analyses on the deformation microstructure as shown in Figure 7. The bright-field (BF) TEM micrograph (Figure 7a) reveals the presence of lamellar deformation twins (DTs). The corresponding selected area electron diffraction (SAED) pattern confirms the typical fcc-twin relationship with the dual spots. High-resolution (HR) TEM imaging in Figure 7b indicates the twin thickness ranges from 2 nm to 10 nm. Stacking faults (SFs) are also observed (Figure 7c,d), and the corresponding SAED pattern (inset of Figure 7d) taken from this region shows the absence of twins or the hcp phase. In low SFE metals, these SFs are a prerequisite for mechanical twinning during deformation [47]. Deformation twins usually promote strain hardening by inhibiting mobile dislocations [39] according to the so-called dynamic Hall–Petch effect where the twins impose a dynamic refinement of the grain interiors, which provide enough barriers against dislocation motion during deformation [48].

Thus, in a case where the twinning propensity is higher and the intrinsic dislocation multiplication is favorable, the plastic strain storage capacity is effectively enhanced by the dislocation–dislocation, dislocation–twin, and twin–twin interactions to maintain strain hardening. On the other hand, an insufficient mobile slip system would effectively raise the intrinsic friction stress and a subsequent high strain hardening rate [44]. However, due to the limited mobile slip systems, the dislocation motion becomes hindered, which affects plasticity. This is detrimental to the effective accumulation of plastic strain such that even though deformation twinning occurs, the early fracture of the sample will occur in 0Al alloys. Hence, even though both the 0Al and 5Al HEAs deform via mechanical twinning, the ease in dislocation nucleation and improved twinning propensities by the Al alloying results in a massive improvement in strength–plasticity synergy.

## 5. Conclusions

The present study investigated the influence of Al alloying on the tensile behavior of an fcc-structured Co-rich HEA at room temperature. The (Co_40_Fe_25_Cr_20_Ni_15_)_100−x_Al_x_ (x = 0 and 5 at.%) HEA series were prepared by a conventional thermomechanical processing route involving homogenization, cold-rolling, and recrystallization annealing. The following conclusions could be drawn:

(1) By alloying with 5 at.% Al, the fcc microstructure of the Co-rich HEA was retained. Annealing at 973 K, 1023 K, and 1123 K resulted in grain sizes ranging from ~2.5 µm to ~5.3 µm for the 0Al samples and ~3.4 µm to 10.6 µm for the 5Al samples.
(2) The relationship between the tensile strength and plasticity followed the classical strength-ductility tradeoff in both alloy samples. As the annealing temperature increased, the tensile strength decreased from 876 MPa to 795 MPa while the plasticity increased from ~14% to 24% for the 0Al samples. Similarly, the tensile strength decreased from 947 MPa to 830 MPa and the plasticity increased from ~36% to 50% as the annealing temperature increased for the 5Al samples.
(3) The addition of the Al significantly modified the fracture mechanism from a dominant quasi-cleavage to a ductile dimple fracture mode. This was attributed to the prolonged strain hardening and the fulfillment of Considere’s criterion for uniform elongation by the 5Al samples.
(4) EBSD analyses of the deformation microstructures revealed both alloys were deformed primarily by deformation twinning. However, the twin fraction for the 5Al alloy was considerably higher relative to the 0Al alloy. The higher twinning propensity by the 5Al sample was further verified by MD simulations, and the significant difference in the plasticity of the alloys was ascribed to the difficulty in slip mobility for the 0Al alloy.

## Data Availability

All data used in this work will be available upon reasonable request.
